# Aspects of Additional Psychiatric Disorders in Severe Depression/Melancholia: A Comparison between Suicides and Controls and General Pattern

**DOI:** 10.3390/ijerph15071299

**Published:** 2018-06-21

**Authors:** Ulrika Heu, Mats Bogren, August G. Wang, Louise Brådvik

**Affiliations:** 1 Department of Clinical Sciences Helsingborg, SE 251 87 Helsingborg, Sweden; Ulrika.Heu@skane.se; 2 Department of Clinical Sciences, University of Lund, Division of Psychiatry; Skåne University Hospital, Baravägen 1, SE-221 85 Lund, Sweden; Mats.Bogren@med.lu.se; 3 Centre of Suicide Prevention, University Hospital Amager, Digevej 110, DK-2300 Copenhagen, Denmark; august.g.wang@gmail.com

**Keywords:** Melancholia, comorbidity, suicide, long-term follow-up

## Abstract

Objective: Additional and comorbid diagnoses are common among suicide victims with major depressive disorder (MDD) and have been shown to increase the suicide risk. The aim of the present study was first, to investigate whether patients with severe depression/melancholia who had died by suicide showed more additional psychiatric disorders than a matched control group. Second, general rates of comorbid and additional diagnoses in the total group of patients were estimated and compared with literature on MDD. Method: A blind record evaluation was performed on 100 suicide victims with severe depression/melancholia (MDD with melancholic and/or psychotic features: MDD-M/P) and matched controls admitted to the Department of Psychiatry, Lund, Sweden between 1956 and 1969 and monitored to 2010. Diagnoses in addition to severe depression were noted. Results: Less than half of both the suicides and controls had just one psychiatric disorder (47% in the suicide and 46% in the control group). The average number of diagnoses was 1.80 and 1.82, respectively. Additional diagnoses were not related to an increased suicide risk. Anxiety was the most common diagnosis. Occurrence of suspected schizophrenia/schizotypal or additional obsessive-compulsive symptoms were more common than expected, but alcohol use disorders did not appear very frequent. Conclusions: The known increased risk of suicide in MDD with comorbid/additional diagnoses does not seem to apply to persons with MDD-M/P (major depressive disorder-depression/Melancholia). Some diagnoses, such as schizophrenia/schizotypal disorders, were more frequent than expected, which is discussed, and a genetic overlap with MDD-M/P is proposed.

## 1. Introduction

Patients with an affective disorder have an increased risk of suicide compared to the general population [1], and 4–6.8% of all persons with an affective disorder have been found to die by their own hand [1,2,3,4,5]. Investigations into suicide victims have shown that 35–59% have suffered from mood disorders [6,7,8], and melancholia seems to predominate in the depressive group [6,7]. 

Several studies have also shown that people who have committed suicide often had more than one psychiatric disorder. Psychiatric comorbidity usually implies a secondary psychiatric diagnosis or alcohol/drug abuse. Comorbidity is common in all mental illness, including depression [9]. The most prevalent psychiatric comorbidity is anxiety disorders [8]. A large study from NCS-R (National Comorbidity Survey-Replication) showed that more than 72% of patients with lifetime major depressive disorder (MDD) also fulfilled the criteria for at least one additional lifetime psychiatric diagnosis, e.g., anxiety disorder (59,2%), substance use disorder (SUD) (24%), and impulse control disorder (30%) [9]. Melancholic patients have been found to have more axis-1 comorbidities and more severe symptoms and suicidal ideations than non-melancholic patients [10]. However, other studies have shown no difference in proportion of personality disorders between non-melancholic MDD and melancholic MDD [11].

Comorbid psychiatric diagnoses have more frequently been found in suicide victims than in non-suicides [12]. Henriksson et al. (1993) found that only 12% of the suicide victims in their study had MDD only, whereas 44% had two psychiatric diagnoses including MDD [13]. Nordentoft et al. found that the cumulative incidence of suicide increased by 3.8–6.6% for each specific mental disorder added [4]. Holmstrand et al. found that the OR for suicide in subjects with one psychiatric diagnosis was 11.8 compared to those who had no psychiatric diagnosis, whereas in subjects with two or more psychiatric diagnoses the OR was 21.0 [14]. Thaipisuttikul found that patients with psychiatric comorbidity had a statistically higher suicidal risk compared with patients without [15]. 

Very few studies have examined depression with melancholic features and suicide. Parker et al. found that subjects with depression with melancholic features had a greater prevalence of suicidal ideation [16,17]. However, a large study from Denmark in 2014, involving over 34,000 patients, showed no differences in suicide risk in patients with psychotic or non-psychotic subtype of depression [18]. To the best of our knowledge, whether psychiatric comorbidity in patients with major depression with melancholic or psychotic features (MDD-M/P) is associated with an increased suicide risk has not been investigated. 

Melancholia is described, not as a specific diagnosis, but as a specifier of MDD according to DSM-5 (Diagnostic Statistical Manual, 5th version [19], where lack of reactivity is one of the most important symptoms, but some refer to it as a qualitatively distinct disorder including psychomotor disturbances [16,17,20]. Mood is a basic dimension of experience of both the world and the self, suggesting an existential significance, and in phenomenology melancholia refers to the disruption of this dimension rather than to a certain number of reportable symptoms [21].

In psychiatric terminology, melancholia refers to a brain disorder that globally alters mood, motor and vegetative functions, thereby colouring all emotional, perceptual and cognitive processes [22]. The clinical manifestations of melancholia are pervasive gloom, apprehension, despair, motor slowing or agitation, disturbed sleep, appetite and libido, emotional non-reactivity, poor memory and concentration, ruminations, retardation and inhibition of thought/speech. Preoccupations, overvalued ideas, delusions and hallucinations, associated with experience of worthlessness, helplessness and guilt, are common.

The present study considers 1206 former inpatients prospectively diagnosed with severe depression/melancholia and followed up from 1956 to 2010, including 116 suicide victims, of which 100 were primary depressives. (It was assumed that patients with melancholia may be genetically predisposed in a different way from those with alcohol use disorders (AUD), who develop depression due to overconsumption of alcohol. This difference may have an impact on suicidal behaviour and suicide risk. Secondary depressives were therefore excluded.) These subjects were compared with 100 matched controls. Case records and multiaxial schedules were evaluated.

### Aim of Study

The first aim of the study was to compare additional and comorbid disorders for suicides and controls to examine whether these disorders were related to an increased suicide risk in MDD-M/P as has been found for other mental disorders, such as MDD.

- Were comorbid/additional psychiatric disorders more frequently found in suicides with MDD-M/P than in controls?
- Did any specific type of additional disorder discriminate between suicides and controls?

A second aim was to find out whether there was a different spectrum of comorbid/additional diagnoses in MDD-M/P as compared MDD (as found in literature):

• How frequent are different psychiatric disorders in addition to MDD-M/P in the total group?

## 2. Materials and Method 

**Figure 1:**
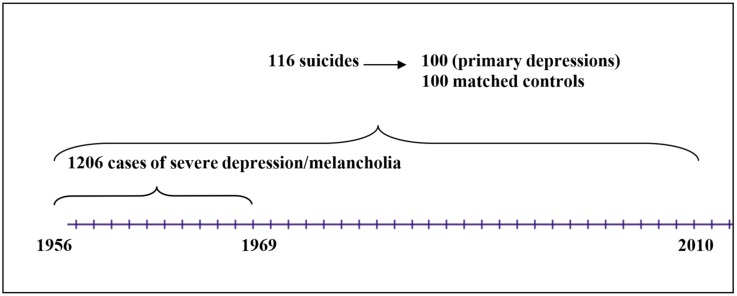
Flow diagram for the sample of patients with severe depression admitted to the Department of Psychiatry, Lund University Hospital.

In the 1950s and 1960s, all inpatients at the Department of Psychiatry, University Hospital, Lund were rated on a multiaxial diagnostic schedule by a senior doctor with at least three years of training at discharge [23]. The schedule comprised one symptomatic or syndromatic and one etiological part. The symptomatic part consisted of 68 items, and increased, mainly by the addition of new items, to 98. The etiological part consisted of 68 items and increased to 74 items. The rater was asked to score symptoms as well as causative factors considered relevant for the individual patient. The rater also marked the main diagnosis, which consisted of the most relevant symptomatic item combined with the most causative item as well as a ”gross syndrome”. This database enabled the selection of patients with a prospectively rated severe depression/melancholia for an investigation into suicide. The design of the sampling procedure is presented in a flow diagram (Figure 1). 

A total of 1206 patients were given this diagnosis (506 men and 700 women). Their mortality was followed-up in three sessions: to 1 January 1984 [24,25], 1 January 1998 [26], and 10 February 2010 [27]. There were 103, 114, and 116 suicides, respectively. Fourteen of these patients had been admitted to the clinic for an AUD before they received the diagnosis severe depression/melancholia and were therefore excluded. (One patient had severe brain damage before index admission, and one was known to the investigator. Those two were also excluded.) This left us with 100 patients who had died by suicides, 44 men and 56 women, with a primary severe depression.

The case records of the suicides and matched controls were prepared for blind evaluation by omitting the last sheet with possible information on the suicide, as has previously been described [27], and a similar procedure was used at second and third follow-up. The controls were randomly selected from a list of the total sample by a research assistant and matched by gender, age, index admission year and time of follow up. If a selected control had died before the suicide victim it was supposed to match, it was substituted by another control. When a patient committed suicide, the case records of the matched controls were cut for further investigation, and therefore covered the same time span in both suicides and controls (a control may survive a suicide with several decades in this longitudinal study and therefore have a too lengthy case record). Up to January 1st, 2014 all but 15 controls had died, all by natural causes (personal communication).

Case-record studies on all 200 patients included validation of diagnoses on the multiaxial rating scale against DSM-IV criteria [28] for MDD-M/P. Mental states were well described in the case records and were matched with criteria for melancholic and/or psychotic specifiers according to DSM-IV, first ‘loss of pleasure’ or ‘lack of reactivity’, second three or more of ‘early morning awakening’, ‘marked psychomotor retardation’, ‘significant anorexia or weight loss’, etc. Mood-congruent psychotic features with delusions or hallucinations whose content is entirely consistent with the typical depressive themes, etc. were considered as MDD with psychotic features (all were mood-congruent).

Good correspondence was found, as 91% of the patients in both the suicide and control group could be shown to meet the criteria for MDD-M/P. 

The mean number of depressive episodes was 3.2 (range 1–32), and average time of follow-up was 14.5 (SD+/−10.7) years. There were 44 men and 56 women in each group. The mean age at first depressive episode was 41 (SD+/−15.07). No significant differences in social class between suicides and controls had been found in a previous study [27].

In the present sample, 20 cases of bipolar disorder were found in both suicides and controls [26]. In all but one case the first switch was from depression to mania/hypomania. In DSM-5, bipolar disorder is considered to be a separate diagnosis, but Taylor and Fink [22] have proposed that bipolar disorder, as well as depressive psychosis, should be included in the melancholic group. We have chosen the latter view, and not considered bipolarity as an additional disorder in the present study, as melancholia is already part of the bipolar disorder in the present sample. Similarly, psychotic disorder was not considered a separate entity. (There were 57 cases of psychosis in the suicide group and 58 in the control group, and 29 patients—14 suicides and 15 controls, had melancholic as well as psychotic episodes in the follow-up.)

Diagnoses additional to MDD-M/P were assessed according to ratings in the case records. They sometimes occurred during the depressive episode and were then comorbid, but they could also occur during another admission. There was sometimes a switch between different types of episodes during the long-term follow-up. These diagnoses were ‘suspected schizophrenia/schizotypal disorder’, ‘organic disorder’ (including dementia), and ‘schizoaffective disorder’. Comorbid symptoms of ‘anxiety’ and ‘obsessive-compulsive symptomatology’ were also rated. Finally, some patients developed AUD. These were secondary to depression, as primary cases were excluded. The additional diagnoses were evaluated in the same way in both the suicide and control groups, but often the description of symptoms was not sufficiently detailed to allow validation against DSM-IV [29]. ‘Anxiety’ was scored on the multiaxial rating scale, and these scores were added to those from the case records. ‘Personality disorders’ had also been scored on the rating scale and were divided into ’normal’ and ’abnormal’.

### 2.1. Statistics

A logistic regression was performed to compare rates of different diagnoses in suicides and controls. Mann-Whitney’s U-test was used to compare number of episodes. 

### 2.2. Ethics

The study was approved by Lund University Medical Ethics Committee in 1985 and 2003.

## 3. Results

### 3.1. Additional Diagnoses in Suicides and Controls

The number of disorders, including the index diagnosis MDD-M/P, is presented in Table 1. Overall, 47% in the suicide group and 46% in the control group had only one disorder. 

A similar number of patients in the suicide and control groups had two disorders (32% versus 33%). Similar numbers were also found in the groups for three, four or more disorders without any significant differences. 

The suicide group had an average of 1.80 psychiatric diagnoses, including the index depression, compared to the control group, which had 1.82. Female suicides had 1.9 episodes versus 2.0 for controls, and the corresponding figures for males were 1.6 versus 1.6. Comorbid/additional disorders did not appear to be a risk factor for suicide in MDD-M/P.

### 3.2. Psychiatric Diagnoses in the Total Sample

The individual disorders apart from MDD-M/P are presented in Table 2. A logistic regression was performed and did not show any significant correlation for any diagnosis.

Schizophrenia/schizotypal disorders, including one case of paranoid schizophrenia, occurred in 9.5% of the total sample. In five cases the episode preceded the depressive episode, and in ten cases episodes succeeded the depression. Three cases were mixed and in one case the schizotypal episode occurred between the depressive episodes.

Comorbid anxiety was common and found in 39.5% of the patients.

A schizoaffective disorder was found in 3.5% of the patients. Organic syndromes, including dementia, occurred in 10% of the patients. OCS was found in 10%, and alcohol use disorders in 4%. Finally, one suicide had a personality disorder according to the multiaxial schedule. 

## 4. Discussion

### 4.1. Main Findings

The present results indicate that comorbid or additional diagnoses did not increase the risk of suicide in patients with MDD-M/P. Less than half of the patients among suicides and controls had only one psychiatric diagnosis, and the average number of diagnoses was 1.8 in both groups. 

The pattern of comorbidity/additional disorders in MDD-M/P appears to be different from MDD, with high rates of schizotypal disorders/schizophrenia, and OCS and AUD were not common.

Most studies show an increased suicide risk with comorbid disorders [12,14]. Data from the Lundby Study [15] showed that each additional disorder increased the suicide risk. The difference between that study and the present one is that the former focused on a community sample with most participants having MDD, so it is not contradictory to the present findings. Comorbid diagnoses often include AUDs. Patients with primary alcohol abuse had been excluded in the present study. A Finnish study showed that 28% of suicide victims with MDD had concomitant alcohol dependence or abuse, regardless of the order of onset of AUD and depression [15]. However, we found few patients who developed AUD in addition to severe depression (4%). According to Parker et al. comorbid alcohol abuse was uncommon in melancholics as well as non-melancholics, 2.2% in both [25], figures the corresponded with the present findings. 

The apparent lack of impact of comorbid/additional disorders on suicide risk in MDD-M/P, as opposed to MDD, could be interpreted as melancholia being a separate entity, as has been proposed by some authors [16]. Melancholia as such appears to have a high impact on suicide risk, and additional disorders do not seem to add much.

Another finding in the present study was that the overall comorbidity was somewhat different from what is usually found in non-melancholic depression. There were high rates of schizophrenia/schizotypal disorders, and obsessive/compulsive symptomatology, but low rates of AUD, regardless of the suicidal outcome.

In the present study anxiety (39.5%) was the most common comorbid diagnosis, which agrees with other studies. Thaipisuttikul et al. suggested in 2014 that almost 21% of patients with MDD had at least one type of anxiety disorder [15]. A large study from NCS-R (National Comorbidity Survey-Replication) showed that over 59.2% of patients with MDD fulfilled the criteria for anxiety disorders [9]. 

The frequency of schizophrenia (9.5%) was much higher than the estimated median lifetime morbid risk of schizophrenia in a systematic review, which was 7.2/1000 persons [29], and the observed 50-year prevalence of schizophrenia in the Lundby population, which was 1.43/100 persons [30]. 

Depressed mood is common both during the prodrome of schizophrenia and after the onset of psychosis. A population-based study of first episode of schizophrenia showed that the most frequent initial symptom of schizophrenia was depressed mood. It was also found that 71% of the subjects with schizophrenia presented with clinically relevant depressive symptoms, and 23% fulfilled the ICD-10 criteria for a depressive episode in their first psychotic episode [31]. Schizophrenia/schizotypal disorders were often found as separate episodes in the present study, and these episodes may precede as well as occur between or succeed the MDD-M/P episodes.

Genetic studies have shown that schizophrenia, bipolar disorder and MDD share genetic risk factors [32,33]. Several molecular genetic studies have focused on common genetics for two or more psychiatric diagnoses, including different aspects of depression. A review from 2013 by Domschke [34] showed several studies that shared potential risk for psychotic depression, schizoaffective disorder, schizophrenia and affective disorder. Chen et al. [35] found a relationship for schizophrenia, bipolar, and MDD. O´Connell et al. [36] identified a genetic association between autism spectrum disorder, schizophrenia, bipolar disorder and OCD. It has also been proposed that negative symptoms are a link between schizophrenia, melancholia, and Parkinson’s disease [37].

It has been suggested that there is a subgroup of MDD that is causally related to schizophrenia [38]. The present study with unexpectedly high rates of schizotypal episodes supports this suggestion, and we propose that melancholia may be such a subgroup. However, the findings need to be replicated in a larger sample of patients with more validated diagnostics.

OCS was quite common in the present sample, 12% versus 8% in suicides and controls, unlike another study where these disorders were rare among persons with MDD [39], and a figure of 4.7% among patients with MDD was found in one study [15]. The risk of developing these symptoms may be higher in MDD-M/P. A correlation between OCS and bipolar disorder has been shown, which indirectly corresponds with the present findings [40]. These disorders have also recently been associated with an increased risk of suicide [41]. However, in the present study, no increased risk of suicide among those with OCS as an additional diagnosis was found.

Depression may represent the first sign of dementia [42], and it has been debated whether depression increases the risk of dementia [43,44]. The rates of organic disorders in the present study are inconclusive, as the age of the subjects varied greatly, and there were few old patients. Furthermore, all patients with organic disorders did not suffer from dementia.

Comorbid diagnoses often include AUD, i.e., alcohol and drug abuse. Patients with primary alcohol abuse were excluded. Secondary alcohol abuse was included (4%). According to Parker et al. the comorbid alcohol abuse was small in melancholics and non-melancholics, 2.2% in both, which corresponds with the findings in the present study [23]. AUD may also be underreported in the case records. Personality disorders were rare, may be due to less inclusive definitions at the time and maybe somewhat less frequent in melancholia. In a previous study; however, brittle/sensitive personality was associated with an increased risk of suicide in the male group [26]. We did not replicate this scoring in the present study.

### 4.2. Limitations and Strengths

One strength was the good correspondence between MDD-M/P and severe depression/melancholia according to DSM-IV in a previous evaluation of the present sample. The long-term follow-up and fairly large sample size are other strengths. However, the additional disorders could not be evaluated against DSM-IV, which is a limitation. All symptoms may not be noted in the case records, which may also lead to an underestimate of symptomatology. AUD is probably underestimated, as there was no systematic inquiry, but this probably does not affect the comparison between suicides and controls, as the evaluation is the same in both groups.

Another limitation is the in-patient sample, which is not fully representative of a community sample.

When estimating the rates of comorbid/additional disorders for the total sample, there is a question about the representativity of control group for the total sample of non-suicides. Although the patients were matched for age and index admission year, some sociodemographic factors may be risk factors for suicide. However, there were similar female/male rates in the total sample (700/506 = 1.38) and in the present controls (56/44 = 1.27). Social group did not discriminate between suicides and controls in a previous study on the same sample [26]. In that study marital isolation at index admission was associated with an increased suicide risk in the female group. However, data before suicide are incomplete and we can therefore not investigate the impact of social factors on suicide. It has for instance been shown that, controlling for the psychiatric, social, and economic predictors of suicide completions, recent divorce increases the odds of death by suicide 1.6 times, compared with 1.3 times for distal divorce [45].

## 5. Conclusions

Comorbid/additional diagnoses were common in the present sample of patients with MDD-M/P. However, there were no increased rates among suicides compared to controls, so there was no indication of increased suicide risk with additional diagnoses. This contrasts to what has been found for MDD. Severe depression/melancholia may have a high suicide risk per se, and additional diagnoses do not appear to increase the risk.

Schizophrenic/schizotypal disorders were unexpectedly frequent, and a common genetic disposition with MDD-M/P, possibly higher than for MDD, is proposed. This finding may be an interesting topic for future research on a larger sample of patients with more systematic diagnostics and genetic testing. Other common disorders were anxiety and OCS. These findings support the view of MDD-M/P being qualitatively different from MDD.

## Figures and Tables

**Table 1:** Number of additional diagnoses apart from MDD-M/P.

Number	0	1	2	3	4	Total
Control	46	33	16	4	1	100
Suicide	47	32	15	4	2	100
Total	93	65	31	8	3	200

**Table 2:** Bivariate calculations and statistics for factors related to suicide.

Factor	Suicide	Controls	P (Pearson chi^2^)
Gender male/female	44/56	44/56	1
Schizophrenia/Non-schizophrenia	09/91	10/90	0.605
Anxiety/non-anxiety	42/58	37/63	0.563
OCS/non-OCS	12/88	8/92	0.480
Organic/non-organic	10/90	17/83	0.214
Schizoaffective/non-schizoaffective	02/98	05/95	0.445
Alcohol/non-alcohol	05/95	03/97	0.721
Number extra diagnoses/no extra	5 categories	5 categories	0.963 (Mann-Whitney test)
Extra diagnosis/no extra	54/46	53/47	1

A logistic regression shows non-significant tendencies for OCS (Obsessive Compulsive Symptomatology) to be related to suicide (P < 0.291) and organic and schizoaffective to disorders to be related to non-suicide (P < 0.158 and P < 0.300, respectively).
